# Case Report: Immune Mediated Necrotizing Myopathy With IgG Antibodies to 3-Hydroxy-3-Methylglutaryl-Coenzyme a Reductase (HMGCR) May Present With Acute Systolic Heart Failure

**DOI:** 10.3389/fneur.2020.571716

**Published:** 2020-11-25

**Authors:** Malik Ghannam, Georgios Manousakis

**Affiliations:** Department of Neurology, University of Minnesota, Minneapolis, MN, United States

**Keywords:** anti-HMGCR antibodies, myopathic, inflammation, systolic heart failure, critical diagnosis

## Abstract

Involvement of cardiac muscle is felt to be very uncommon in anti-HMGCR myopathy, and therefore early cardiac evaluation is not considered a high priority for this condition. We herein present the case of a 72 year-old woman admitted due to dyspnea and orthopnea, who, in retrospect, suffered from proximal more than distal muscle weakness for 3 months prior to admission. She was found to have acute systolic heart failure. Serologic testing showed positive 3-hydroxy-3-methylglutaryl-coenzyme A reductase (HMGCR) IgG antibodies, and muscle biopsy showed necrotizing myopathy. No alternative explanation for heart failure was found. Despite immunotherapy and symptomatic treatment, she died from multiorgan failure. Our study suggests that heart failure in anti HMGCR myopathy may not be as rare as previously thought, and therefore early cardiac evaluation should be considered in patients with this diagnosis, to minimize morbidity and mortality.

## Background

Anti-HMGCR myopathy is a rare form of immune mediated necrotizing myopathy that was initially described in patients with a history of statin exposure, although up to one third of affected patients report no such history (1). It is characterized by progressive limb-girdle muscle weakness with marked creatine kinase elevation (CK), commonly in the range of 1,000–20,000 IU/L (2). Clinical features also include fatigue and myalgia (20–60%), dysphagia (16–30%) and truncal weakness (2). Interestingly, some patients may present with a very indolent, chronic course, mimicking limb-girdle muscular dystrophy, and few may have additional extra-muscular features such as rashes mimicking dermatomyositis (Gottron's and heliotrope) (3, 4). Fewer than 5% of patients develop interstitial lung disease (5). We report a case of acute systolic heart failure secondary to anti-HMGCR myopathy, which is not a typical feature of this condition.

## Presentation

A 72 year-old female with a past medical history of childhood poliomyelitis with residual left hemiplegia, and hyperlipidemia on simvastatin, was admitted to our hospital due to dyspnea and orthopnea. She reported a 3 month history of progressive, painless, right-sided proximal more than distal weakness of upper and lower extremities, which were not previously affected by polio. Three months prior to admission she required a cane to ambulate, and 3 weeks prior to admission she began using a wheelchair. She denied any sensory symptoms other than tingling at the toes on her right foot. A full physical examination performed by her primary care physician 3 months prior to admission showed no abnormal cardiac signs, specifically normal S1, S2 sounds, lack of jugular venous distention, pedal edema, rales, or rhonchi.

Examination on admission showed that she was afebrile, but tachypneic (25 breaths/min) and tachycardic with heart rate 110–125 per minute. Blood pressure ranged between 101 and 150 mm Hg systolic and 53–103 diastolic. Jugular venous pressure was 10 cm H_2_O. Auscultation of lungs revealed bibasilar rales. There was no pedal edema. Neurological examination showed flaccid left hemiplegia (Medical Research Council (MRC) 0/5 strength, except for 3/5 left knee extension) and marked muscular atrophy. Right shoulder abduction and hip flexion were 2/5, right elbow flexion, extension, wrist extension, knee extension, and foot dorsiflexion were 4/5, right knee flexion was 3/5, and distal right upper extremity muscles were normal. The rest of her neurological exam was unremarkable except for absent reflexes on the left side.

**Figure 1:**
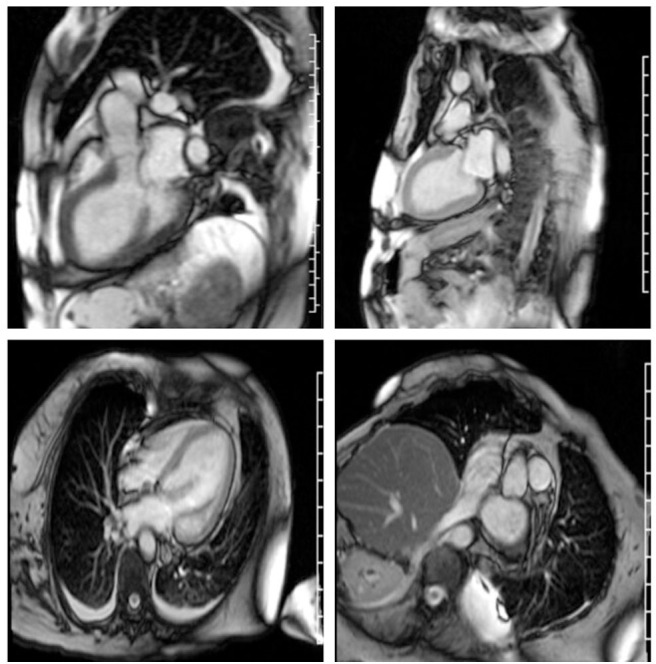
Severe cardiomyopathy. **Left** ventricular ejection fraction was calculated at 10–20%. **Right** ventricular ejection fraction was normal. There is no myocardial fibrosis or enhancement.

Echocardiography 2 days prior to admission showed ejection fraction of 30%, and a week later 14%, and severe diffuse hypokinesis. Cardiac MRI confirmed those findings; there was no valvular disease and no late gadolinium enhancement (Figure 1). N-Terminal Pro BNP was 5,951 pg/ml. CK was 3,802 U/L. Aldolase was 29.1 U/L. White blood count was 28,700/ul. Sedimentation rate was 79 mm/h. C-Reactive Protein was 6.5 mg/dl. Troponin was elevated but peaked at 1.935 ug/l. Serum myositis antibodies including Jo1, PL7, PL12, EJ, OJ, SRP, Mi-2, NXP2, TIF1-gamma, MDA5, SAE1, were negative. Thyroid stimulating hormone was normal. CT angiography of chest was negative for pulmonary embolism but showed pulmonary edema with bilateral pleural effusions. Cardiology was consulted and working diagnosis was non-ischemic cardiomyopathy related to myositis. Diuresis with intravenous furosemide 40 mg twice a day and captopril 12.5 mg three times a day was recommended. Patient's dyspnea and orthopnea significantly improved after diuresis. MRI of the right shoulder with contrast revealed muscular and deep fascial edema as well as mild diffuse enhancement consistent with myositis. Similar findings were appreciated at the posterior paraspinal muscles on a lumbar spine MRI. Infectious workup for myositis was negative including Interferon Gamma Release Assay, Hepatitis B Surface antigen, Adenovirus PCR, Enterovirus PCR, Echovirus, Coxsackie, HTLV I, II, hepatitis C, and HIV antibodies, and respiratory virus Panel. The patient was empirically treated with 1 gram of intravenous methylprednisolone daily for 3 days followed by 50 mg oral prednisone daily with a plan for right deltoid muscle biopsy. EMG showed large, long duration motor unit potentials with reduced recruitment in all muscles of the right upper and lower extremities, consistent with chronic neurogenic disorder like poliomyelitis. However, extensive abnormal spontaneous activity was also detected, including fibrillations, complex repetitive, and occasional pseudomyotonic discharges, which were not typical for remote poliomyelitis, and suggested a superimposed myopathy with membrane irritability.

The patient's condition continued to deteriorate with more weakness and dyspnea. Furosemide was increased to 60 mg twice a day and she was started on intravenous immunoglobulin, 2 grams/kg over 3 days. IgG antibodies to 3-hydroxy-3-methylglutaryl-coenzyme A reductase (HMGCR) measured by ELISA assay (ARUP Lab) came back strongly positive (>200 units, normal is 0–19), consistent with the diagnosis of necrotizing myopathy. Her condition continued to deteriorate, and she became acutely encephalopathic due to hypercapnic respiratory failure, requiring intubation. She became hyponatremic, and therefore furosemide was stopped. Captopril was increased to 50 mg three times a day.

**Figure 2:**
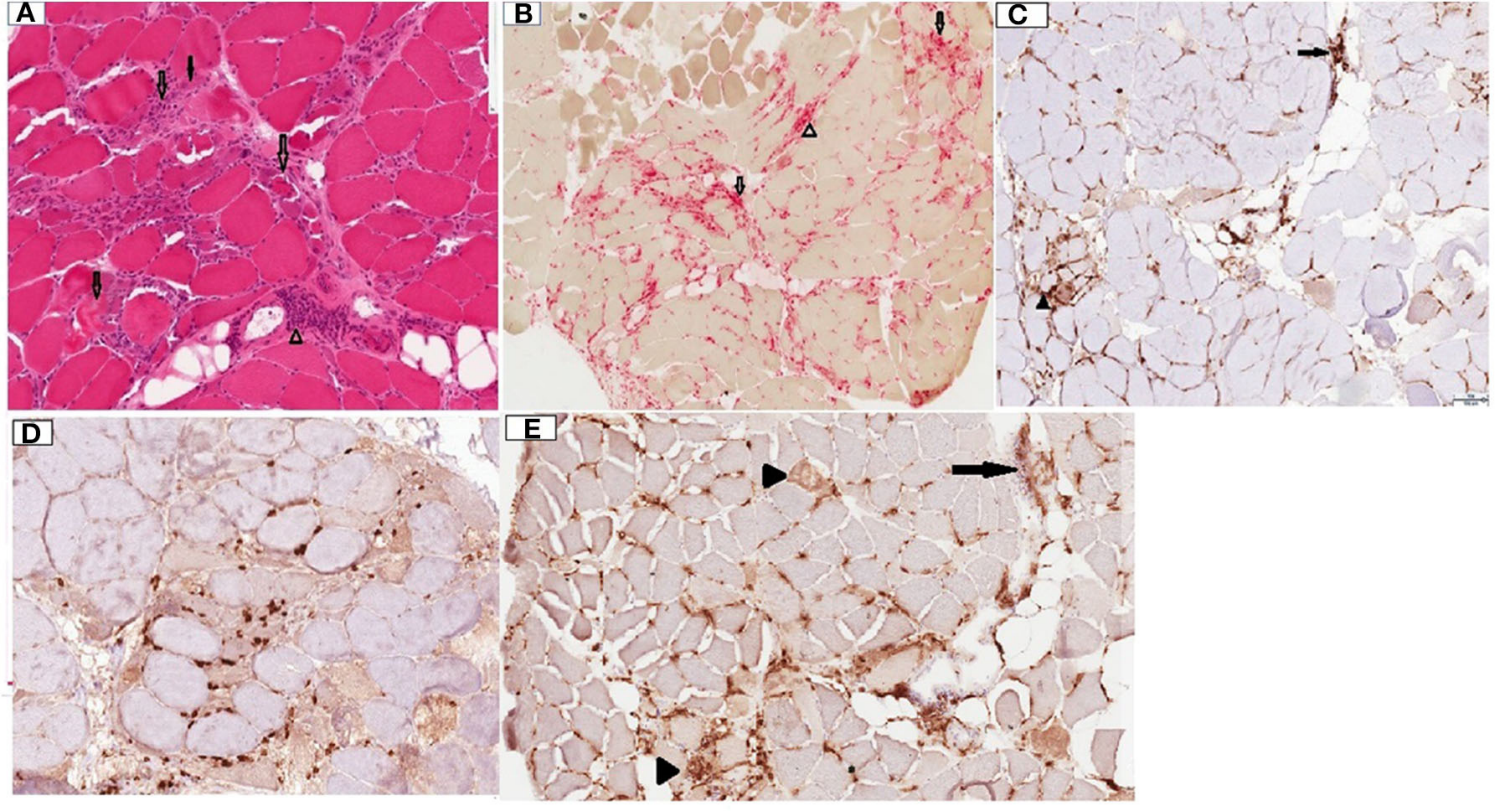
Deltoid muscle biopsy, transverse sections. **(A)** Hematoxylin and Eosin (H&E) stain, 10x. Note the presence of multiple fibers at different stages of necrosis (transparent arrows), regenerating basophilic fibers (solid arrow) and lymphocytic inflammation in perimysium surrounding a blood vessel (arrowhead). **(B)** Acid phosphatase stain, 4x. Note necrotic fibers (transparent arrows) and perimysial histiocytic inflammation (arrowhead). **(C)** CD4 stain, 10x. Note mild endomysial (arrowhead) and perivascular (arrow) staining. **(D)** CD8 stain, 10x. Scattered positive endomysial cellularity. **(E)** CD68, 10x. Note prominent expression in necrotic fibers (arrowheads) and perimysial/perivascular regions (arrow). CD20 staining was negative (not shown).

Patient was given rituximab 1,000 mg intravenously, with plan to repeat the same dose in 2 weeks. A deltoid muscle biopsy revealed multifocal fiber necrosis and regeneration, with occasional foci of perimysial and perivascular inflammation, which was consistent with the diagnosis of acute necrotizing myopathy (Figure 2). The patient clinical course deteriorated further with multiorgan failure. After a family meeting, the patient was placed on comfort care orders, and she died shortly after palliative extubation, 1 month after the hospital admission. Autopsy was not performed.

## Discussion

In the presence of high pretest probability like muscle weakness, elevated CK or typical biopsy findings, detection of anti-HMGCR antibody by ELISA assay has high specificity for diagnosing anti-HMGCR myopathy. Importantly, those antibodies are not detected in patients with myalgia or reversible toxic rhabdomyolysis related to statins (2). Muscle biopsy typically reveals patchy myofiber necrosis, and regeneration. While inflammatory infiltrates are much less prominent than dermatomyositis or polymyositis, more than half of patients may have perimysial pathology, with thickening, fragmentation, and histiocytic inflammation, and 30% may show occasional perivascular lymphocytic collections, similarly to our case (6).

The first step of treating anti-HMGCR myopathy is to stop statin drugs, but aggressive immunotherapy is required to achieve remission of the disease. While randomized controlled trials to guide treatment are lacking, expert opinion suggests that a multi-drug regimen including intravenous immunoglobulin, corticosteroids, and a steroid sparing immunosuppressive agent like azathioprine, mycophenolate, methotrexate or rituximab should be offered. Given that passive transfer experiments in mice showed that the antibodies are directly pathogenic (7), removal of the antibodies with plasma exchange may also be considered to produce a transient improvement. While rare patients may experience spontaneous remissions, most patients require some immunotherapy to maintain remission, and relapses upon discontinuation are not uncommon (2, 8).

Cardiac involvement is a well-known feature of other immune myopathies, such as polymyositis, dermatomyositis (PM & DM), and anti-SRP antibody necrotizing myopathy (5, 9). PM and DM can exhibit a wide spectrum of cardiac abnormalities, including pericarditis, myocarditis, conduction system abnormalities and mitral valve prolapse (10). Cardiomyopathy with heart failure is the most common cardiac abnormality in PM, it is one of the main prognostic factors for this condition and can cause death in 10–20% of patients (9, 11). The existing literature suggests that cardiomyopathy is not a typical feature of anti-HMGCR myopathy (2). Our findings, in addition to two previous case reports (12, 13), indicate that acute systolic heart failure can occur in HMGCR myopathy, and since this is a serious, treatable complication, early diagnosis is critical.

Our study has some limitations, making the association of the cardiac disease with the necrotizing myopathy putative, rather than fully proven. There was no evidence of pre-existing heart disorder, based on recent pre-admission physical examination, and alternative causes, including infections, thyroid disease, and illicit drug use were eliminated by serological testing and history. However, a coronary angiogram was not performed, therefore ischemic cardiomyopathy cannot be completely excluded, although the findings of global hypokinesis on echocardiogram and cardiac MRI are not typical for ischemia, where regional wall motion abnormalities are frequently encountered. Second, the MRI findings did not meet the Lake Louise criteria of myocarditis (14), specifically there was no edema on T2 and no late gadolinium enhancement. The sensitivity of those imaging criteria is 80%. Third, there was no autopsy or endomyocardial biopsy performed to evaluate the histopathologic changes of the myocardium and compare to the ones identified on the skeletal muscle biopsy. Fourth, the patient did not improve with immunotherapy, although severe cases of necrotizing autoimmune myopathy may not always respond to treatment. Nevertheless, our case, added to the two previously reported cases with similar phenotype, suggests that heart failure in anti HMGCR myopathy may not be as rare as previously thought, and therefore early cardiac evaluation with electrocardiogram and imaging should be considered in patients with this diagnosis, to minimize morbidity and mortality.

## Data Availability Statement

The original contributions presented in the study are included in the article/supplementary materials, further inquiries can be directed to the corresponding author/s.

## Ethics Statement

Written informed consent was obtained from the individual(s) for the publication of any potentially identifiable images or data included in this article.

## Author Contributions

MG and GM were responsible for the clinical management of the patient. MG was responsible for drafting and editing of the manuscript. GM participated in critical revision of the manuscript for intellectual content. All authors read and approved the final manuscript.

## Conflict of Interest

The authors declare that the research was conducted in the absence of any commercial or financial relationships that could be construed as a potential conflict of interest.
